# Perspective on optical imaging for functional assessment in musculoskeletal extremity trauma surgery

**DOI:** 10.1117/1.JBO.25.8.080601

**Published:** 2020-08-31

**Authors:** Ida L. Gitajn, Gerard P. Slobogean, Eric R. Henderson, Arvind G. von Keudell, Mitchel B. Harris, John A. Scolaro, Nathan N. O’Hara, Jonathan T. Elliott, Brian W. Pogue, Shudong Jiang

**Affiliations:** a Dartmouth-Hitchcock Medical Center, Department of Orthopaedics, Lebanon, New Hampshire, United States; b University of Maryland, Orthopaedic Associates, Baltimore, Maryland, United States; c Brigham and Women’s Hospital, Department of Orthopaedic Surgery, Boston, Massachusetts, United States; d Massachusetts General Hospital, Department of Orthopaedic Surgery, Boston, Massachusetts, United States; e University of California, Irvine, Department of Orthopaedic Surgery, Orange, California, United States; f Dartmouth-Hitchcock Medical Center, Department of Surgery, Lebanon, New Hampshire, United States; g Dartmouth College, Thayer School of Engineering, Hanover, New Hampshire, United States

**Keywords:** optical imaging, bone perfusion, orthopaedic surgery, fluorescence imaging, indocyanine green

## Abstract

**Significance:** Extremity injury represents the leading cause of trauma hospitalizations among adults under the age of 65 years, and long-term impairments are often substantial. Restoring function depends, in large part, on bone and soft tissue healing. Thus, decisions around treatment strategy are based on assessment of the healing potential of injured bone and/or soft tissue. However, at the present, this assessment is based on subjective clinical clues and/or cadaveric studies without any objective measure. Optical imaging is an ideal method to solve several of these issues.

**Aim:** The aim is to highlight the current challenges in assessing bone and tissue perfusion/viability and the potentially high impact applications for optical imaging in orthopaedic surgery.

**Approach:** The prospective will review the current challenges faced by the orthopaedic surgeon and briefly discuss optical imaging tools that have been published. With this in mind, it will suggest key research areas that could be evolved to help make surgical assessments more objective and quantitative.

**Results:** Orthopaedic surgical procedures should benefit from incorporation of methods to measure functional blood perfusion or tissue metabolism. The types of measurements though can vary in the depth of tissue sampled, with some being quite superficial and others sensing several millimeters into the tissue. Most of these intrasurgical imaging tools represent an ideal way to improve surgical treatment of orthopaedic injuries due to their inherent point-of-care use and their compatibility with real-time management.

**Conclusion:** While there are several optical measurements to directly measure bone function, the choice of tools can determine also the signal strength and depth of sampling. For orthopaedic surgery, real-time data regarding bone and tissue perfusion should lead to more effective patient-specific management of common orthopaedic conditions, requiring deeper penetrance commonly seen with indocyanine green imaging. This will lower morbidity and result in decreased variability associated with how these conditions are managed.

## 1 Introduction

A reliable assessment of tissue viability is critical to effectively treating patients who sustain traumatic extremity injury. Despite their relatively inert appearance, bone, tendons, and ligaments are, physiologically speaking, highly active. Bones, specifically, receive ∼10% of the entire cardiac output of blood volume.^1^ Dysvascular tissues have limited potential to regenerate, heal, or fight infection due to insufficient delivery of inflammatory cells, growth factors, osteoprogenitor cells, endogenous immune cells, and antibiotics. It is clear that, in orthopaedic injury, nonviable or poorly perfused bone and soft tissue inhibits healing and increases the risk for infection. Because of this, assessing bone and soft tissue viability is critical to making effective treatment decisions, and the comparative translucency of these tissues to red and near-infrared light makes optical methods feasible.

## 2 Current Paradigm and Challenges

To date, the eyes and hands of the surgeon remain the dominant “imaging modality” used to make decisions regarding the health of soft tissue or bone. Methods currently used to assess tissue and bone perfusion are subjective and entail non-quantitative clinical cues. These techniques include clinical judgement based on the color and swelling of skin, presence of skin wrinkles, color, and turgor of deep tissues, measurements of prior cadaveric studies (for example, regarding perfusion of the meniscus in the knee),^2^^,^^3^ extent of soft tissue stripping off bone, and the “paprika sign” (defined as scattered pinpoint bleeding on bone).^4^^,^^5^ The subjective or imprecise nature of these assessments leads to substantial variation in treatment and a lack of advancement in objectively driven treatment protocols.

Imaging blood perfusion to soft tissue and bone can be done using traditional imaging modalities, such as positron emission tomography/computed tomography (PET/CT)^6^^,^^7^ and contrast-enhanced magnetic resonance imaging (MRI).^8^ These imaging modalities provide detailed and accurate information of the fractured or diseased bony and soft tissue anatomical structure, yet they have issues with limits to spatial resolution and artifacts from metallic implants. The logistical limitations are the cost recovery, the need to schedule them as a separate procedure, and the limits to using them for routine real-time assessment or repeated re-examination.^6^^,^^7^ MR-based techniques are limited in orthopaedic patients due to metal artifact and signal dropout in association with metal implants. A limited number of studies have used PET/CT or MRI to measure blood flow to bone for surgical indications, including infection, osteomyelitis, and nonunion.^8^^–^^13^ However, there is limited to no access to these imaging methods in real-time in the operating room. At the present time, fluoroscopy and plain radiographs are used regularly in the operating room to visualize bones and assess fracture alignment and/or hardware placement. In contrast to these radiologic modalities, optical imaging can obtain metabolic information that provides physiological abnormities of the tissue on microvascular perfusion and/or bone and tissue viability. The main limitation of optical imaging is the inability to achieve both high spatial resolution and high tissue penetrance: any added value, then, is either in high-resolution imaging at low penetrance or more macroscopic assessment of tissue function at a more moderate to medium penetrance. Thus, optical imaging can be a unique complementary imaging modality to the conventional imaging modalities.

## 3 Potential Optical Imaging and Sensing Needs

### 3.1 Surgical Timing/Skin Assessment

High energy fractures are frequently associated with significant insult to the soft tissues surrounding bone. Historically, immediately performing open reduction and internal fixation of injuries such as tibial plafond, tibial plateau, and calcaneus fractures has been associated with unacceptably high rates of infections and wound complications.^14^^–^^17^ Based on these experiences, staged protocols in which fractures were temporized in traction, splints, or external fixators until the soft tissue envelope had recovered enough to undergo open reduction internal fixation were introduced.^18^^–^^21^ Unfortunately, no objective definitive clinical signs exist to determine the timing for definitive fixation.^22^ Surgeons frequently use clinical clues such as the presence of skin wrinkles or epithelialization of fracture blisters, but there are no evidence-based objective signs or thresholds to indicate that the soft tissues are appropriate for a surgical incision and open fracture fixation. Staged protocols have resulted in decreased wound complications; however, they have recently been challenged with a resurgence of early fixation for a variety of reasons. For example, there are valid concerns regarding quality of reduction (which becomes increasingly difficult the longer the delay to internal fixation), increased operative time, increased healthcare costs, and pin site infections associated with the use of external fixators, and these have led a number of surgeons to question whether acute definitive internal fixation might be more appropriate in certain patients. While this view is supported by multiple recent studies showing that fractures treated within 72 h of surgery have comparable outcomes compared with staged fixation,^23^^–^^25^ these studies were all retrospective reviews, and the criterion for early fixation was surgeon or facility dependent. It is clear that not all soft tissues are appropriate for acute fixation. Therefore, an objective tool that could effectively measure the soft tissue recovery with thresholds identified in association with appropriateness for definitive internal fixation may significantly improve the current practice. Optical imaging tools that image tissue perfusion of indocyanine green (ICG) have longstanding success regarding assessment of soft tissue viability and perfusion in the setting of plastic surgical indications such as free osseous flap perfusion^26^^,^^27^ or breast reconstruction.^28^^,^^29^ They are, therefore, well suited for this application.

### 3.2 Assessing the Viability of Deep Tissue in Traumatic Wounds

High-energy open fractures, with bone and deep soft tissue exposed to environmental contamination, can be at an extraordinarily high risk of a complication, such as infection and nonunion, occurring in 10% to 60% of patients.^30^^–^^36^ Complications convert a difficult 6-month recovery into a several-year (or longer) recovery that may include unplanned surgical procedures, prolonged morbidity, loss of function, and loss of limb. Bone and soft tissue devitalization is believed to be a critical determinant in the development of complication following traumatic injury. Deficient perfusion or blood flow prevents delivery of inflammatory cells, growth factors, osteoprogenitor cells, endogenous immune cells, and systemically delivered antibiotics,^37^^–^^43^ which limit the potential of the injured extremity to regenerate, heal, or fight infection. Because of this, the cornerstone of management of these injuries includes surgical debridement of all devitalized bone and soft tissue. However, the subjective clinical signs (such as color, turgor, and soft tissue stripping) remain the only signs surgeons can currently use to evaluate bone or soft tissue perfusion or viability. These highly subjective signs lead to substantial variation in the extent of debridement and a high potential for either under- or overdebridement, both of which have potentially catastrophic downstream consequences. What is needed is an intraoperative system that can assess vascular perfusion of bone in the surgical field in real-time, which could guide surgeons in the right amount of bone and soft tissue to debride. Although there are challenges in association with the three-dimensional (3-D) nature of traumatic wounds and open fractures, optical imaging techniques^44^^,^^45^ are uniquely suited to collect data on the inflow/outflow kinetics of complex tissues.

### 3.3 Assessing Perfusion of Deep Tissue in Infection

In the setting of bone infection, or osteomyelitis, it is clear that there are two interconnected problems with regards to effective treatment: (1) location and debridement of all devitalized or poorly perfused bone and (2) location and debridement of all infected bone. In the current paradigm, MRI may be used to assess the extent of bony edema on T2-weighted sequences and marrow replacement on T1-weighted sequences when there is not pre-existing hardware in place. However, more commonly, metallic hardware prevents MRI from providing useful information due to signal dropout around metallic implants. Furthermore, there is not a one-to-one relationship between either vascular perfusion or microbial infiltration in bone and bony edema on T2-weighted sequences or marrow replacement on T1-weighted sequences. The standard of care is otherwise removal of hardware, surgical debridement, and, in the setting of unhealed fracture, staged fracture stabilization using either antibiotic coated or non-antibiotic coated implants. Surgical debridement is carried out using the previously described signs including the color of bone/tissues, turgor, and “paprika sign” or using a burr to assess for bony bleeding. The failure rate of treatment of these issues is unacceptably high, resulting in reoperation and/or amputation 30% of the time in the setting of fracture or fusion.^46^^–^^48^ Because, to date, there are no methods available to measure bone perfusion or bacterial penetrance *in vivo*, there is very little understanding of which of these variables is necessary to guide debridement. Further, there are no intraoperative tools to assess how effective performance is in these debridement procedures. Similar to issues associated with evaluating vascular perfusion of open fractures, the 3-D nature of infected wound beds presents challenges. However, optical imaging systems are uniquely suited to assess perfusion of bone and deep soft tissues in operating room settings in real-time.^27^^,^^44^^,^^45^^,^^49^

### 3.4 Assessing Soft Tissue for Healing Potential

All injured tissue requires adequate perfusion to deliver growth factors necessary for healing. This is true for skin and bone (described above) as well as soft tissue injuries, such as ligament ruptures, meniscal tears, and cartilage injury. This is the rationale for the high failure rate of primary repair of the anterior cruciate ligament (ACL). This unacceptable failure rate led to the abandonment of primary repair in favor of ACL reconstruction using autograft or allograft such as patellar tendon or hamstring.^50^^–^^56^ However, ACL reconstruction is not without issues, and some continue to advocate for primary repair to preserve the native ligament, which maintains proprioception^57^^,^^58^ and prevents complications in graft harvesting, tunnel widening, and revisions.^59^^,^^60^ These issues with reconstruction have led to methods to support a primary repair, including the design of an extracellular matrix scaffold activated with the patients’ blood as an alternate way to deliver growth factors and promote tissue healing.^61^ Several authors have noted that the location of the tear may play a role in outcomes following primary repair, with primary repair of more proximal tears performing better than mid-substance or distally based ACL tears.^62^^–^^67^ Likely, this differential rate of healing of a primary ligament repair between proximally located and other tears is associated with the vascular perfusion of the ligament. Given this, if there was a way to assess vascular perfusion and therefore healing potential arthroscopically, it may be possible to preserve the native ligament in appropriate patients and more effectively manage this increasingly common injury.

Similarly, the standard treatment for a meniscal tear is based on cadaveric studies evaluating the vascular penetrance or vascular perfusion of the meniscus.^2^^,^^3^^,^^68^ From these cadaveric studies, the meniscus was divided according to vascularity into red–red (well perfused), red–white (marginally perfused), or white–white (poorly perfused) zones.^2^^,^^3^^,^^69^^,^^70^ This classification, based on perfusion assessed in cadaveric menisci, is used to indicate whether there is adequate perfusion to allow the meniscus to heal after repair versus location where perfusion is not adequate and whether the area with the meniscal tear should be debrided or removed surgically. However, it is clear that meniscus preservation is the first choice when there is adequate perfusion to prevent the risk of secondary osteoarthritis.^71^^–^^74^ Furthermore, several papers have noted that there is variability in the extent of meniscal perfusion in association with age and other patient-specific variables and that the zones described above are not universally accurate.^75^^–^^80^

In both of these injury types, as well as other soft tissue orthopaedic injuries, the translation of optical imaging techniques to an arthroscope could inform appropriate surgical management (repair versus reconstruction or debridement) in a patient-specific manner rather than basing this decision on vascular perfusion of the average cadaveric specimen. This advancement could simultaneously expand the number of patients eligible for repair, reduce failure rates, and provide both patients and surgeons with reassurance that the correct surgical procedure was performed.

## 4 Optical Tools Utilized to Date

When optical imaging is used for maximum benefit, light of different wavelengths or different interaction mechanisms can be used to capture biophysical changes in the tissue, occurring in the vascular as well as extravascular and cellular matrix compartments.^81^^–^^83^ The key to most of the applications, as with all imaging systems, is to have a good biophysical understanding of the compartmentalization of the endogenous or exogenous chromophores that contribute to each image so that the images can be interpreted appropriately. When compared with conventional medical imaging modalities, such as MRI, PET, or x-ray CTCT, optical imaging detects changes in light reflectance, absorption, and scattering of the tissue and thus offers advantages of nonionizing, low-cost, and portable imaging for routine, low-cost, longitudinal monitoring of response to therapy or during human surgery.^81^^–^^83^ The logistics of use of optical imaging, being real time and at the point of care, make it more comparable to C-arm x-ray or ultrasound. Therefore, any determination of the value of optical imaging in tissue injury care needs to be done within the context of what information can be assessed at the point of surgery decision making.

The challenge of applying optical tools to bone and soft tissue assessment has been the high scattering nature of both tissues, limiting the spatial depth dependence of the information. Clearly surface camera-based imaging tools interrogate a range of depths in the millimeter to centimeter range,^45^ with rapidly decreasing sensitivity and spatial resolution with small increases in depth. So, the two major attractions to optical tools come in the categories of one of three rough regimes:

(1) microscopic imaging and sensing (micron sampling depth and spatial resolution),
(2) surface imaging during surgery (micron-mm sampling depth and spatial resolution),
(3) deep tissue sensing during surgery (mm-cm sampling depth and spatial resolution).

These regimes are defined by the type of tool used and dictate what information can be obtained during imaging. The matching of the capabilities of each tool with the needs in each application are essential to evaluate.

Table 1 presents an overview of the tools that have been tried in soft and hard tissue trauma and disease studies. The engineering aspect of the contrast mechanism is used to categorize the tools in column 1. However, in terms of utility and function for trauma surgery, it is likely much more functional to consider the depth of interaction, column 3, because this is the factor that will determine what biomedical information the tool can supply. For example, optical coherence tomography (OCT) has been widely used^91^^,^^92^ in bone imaging but with penetrance on the order of <1  mm, thereby allowing its use only for largely research studies and unlikely as a major surgical tool. Similarly, ultraviolet (UV) fluorescence imaging provides information bone necrosis, and the addition of antibiotics has been shown to enhance the ability of this fluorescence to provide diagnostic value about osteonecrosis. However, the penetrance of both UV and antibiotic fluorescence to just tens to hundreds of microns (μm) does limit their use to situations in which the imaging is directly on the bone surface in question.

**Table 1:** Listing of optical imaging tools utilized in soft and hard tissue trauma and disease studies, categorized by the type of contrast, depth regime, and a brief strengths and weaknesses list.

Contrast mechanism	Probe technology	Depth regime	Strengths/weaknesses	References
Endogenous signals	UV fluorescence	μm	No exogenous contrast needed;	84, 85
			assessment of bone necrosis;	
			superficial tissues only.	
	Scatter, dichroic, polarization, OCT	μm to 1 mm	No exogenous contrast needed;	86–92
			high spatial resolution;	
			matrix structures and flow;	
			superficial tissues only.	
	NIRS (absorption and scattering)	Several mm to cm	No exogenous contrast needed;	93–96
			functional information of hemodynamics and tissue oxygenation;	
			need to scan probe over region.	
	NIR imaging (reflection)	Several mm	No exogenous contrast needed;	97, 98
			3-D-model-based surface-shape tracking.	
	Raman, NIR-hyperspectral and infrared (IR)	μm to cm	High molecular specificity;	99–109
			very low signal levels;	
			specificity/sensitivity tradeoff.	
	Terahertz (THz)	μm	Superficial sensing;	110, 111
			largely water content based.	
Contrast agents	Antibiotics	μm	Sensing of osteonecrosis or bone growth; well-tolerated;	112–116
			multiday use for contrast.	
	Fluorescence recovery after photobleaching	μm to mm	Intercellular solute flow sensing; used with standard contrast agents;	117–120
			superficial imaging assessment.	
	ICG	Several mm to cm	Vascular/tissue perfusion;	27, 121, 122
			often used qualitatively but can be provide a binary diagnostic;	
			can provide flow kinetics.	
	Molecular contrast and nanoparticles	μm to cm	Matrix and biology targeting;	123–126
			limited to largely preclinical to date, although evolving rapidly.	
Hybrid imaging modalities	Optical/ICG	Several mm to cm	Commercially available and growing in usage;	27, 121, 122
			primarily used for soft tissue due to higher perfusion.	
	Photoacoustic/optoacoustic	Several mm to cm	Primarily studied in soft tissues;	127, 128
			some potential for cancellous bone;	
			high degree of image artifacts.	
	X-ray/Raman	Several mm to cm	High potential for chemical specificity and contrast agents;	129
			very low signal levels.	

In terms of molecular information about bone, perhaps no other measure has the potential to provide high feature information than Raman spectroscopy.^99^^–^^105^ The measures of bone regrowth have been well studied, and measurements through even centimeter thick tissues have been demonstrated with spatially offset Raman or Raman near-infrared (NIR) tomography.^106^^,^^109^ However, practical issues in the very low signal strength and the specificity of the signal have limited the routine application of this technique to research studies to date. More exotic methods such as terahertz imaging have been tried, although their specificity to biological information beyond water content of the tissue is unclear and they are constrained to fairly superficial tissue thicknesses (μm to mm).

At the other end of the penetrance scale (mm to cm), near-infrared spectroscopy (NIRS) can be used through several centimeters of tissue, albeit with very limited spatial resolution, for deep tissue sensing of absorption and scattering features. Time-resolved NIRS, which measures photon time-of-flight allowing for selection of late-arriving photons to increase depth sensitivity, may offer improvements in resolution and quantification and has been used recently in joint imaging.^96^ So, while NIRS has its limitations, it is likely a more useful tool for macroscopic tissue assessment and is largely limited to the measurement of blood, oxygen saturation, water, and lipid content of the tissue or scattering features related to the tissue matrix composition. Thus, it can be used in conjunction with ICG as a dynamic contrast-enhanced NIRS.^130^ Future work might examine the value of NIRS for osteonecrosis assessment or flow. The assessment of blood flow can be done microscopically with OCT imaging. However, macroscopic bone blood flow has been one of the challenges. Recently, the flow assessment of imaging the fluorescence from ICG has been shown to provide predictive value for flow and even potentially differentiate between endosteal and periosteal flow.^27^^,^^44^^,^^45^^,^^49^

Perhaps the most promising tools are ones in which there is a hybrid approach to imaging information, so the tool is not being evaluated as a single diagnostic, but rather is complementary to existing clinical tools or as a combination of more than one tool. The most obvious of these is the use of ICG fluorescence imaging in which it has been added onto color laparoscopy imaging, and the two can be codisplayed on a video monitor for the surgeon to use.^131^ This technique has high penetrance, with several commercial systems approved for use.^132^ The creation of new molecular-specific optical contrast agents is evolving rapidly with many clinical trials ongoing. These should be eventual contrast agents for fluorescence-guided surgery in trauma, even if many of the initial studies are devoted to diseases such as cancer or cardiovascular disease.

Photoacoustic imaging exists as a hybrid optical-ultrasound tool, which can in some cases provide both optical contrast as well as ultrasound images, although like ultrasound it has its strengths in soft tissue imaging and can have large artifacts from the interfaces between hard and soft tissues or voids within either tissue. Still, the photoacoustic mode of imaging vascularity and even flow can be significant and provide exquisite detail on vascular patterns and function when deployed appropriately.^127^^,^^128^^,^^133^ The clinical utility of these systems is evolving rapidly, and evaluating their use in trauma surgery should be a high priority.

**Fig. 1:**
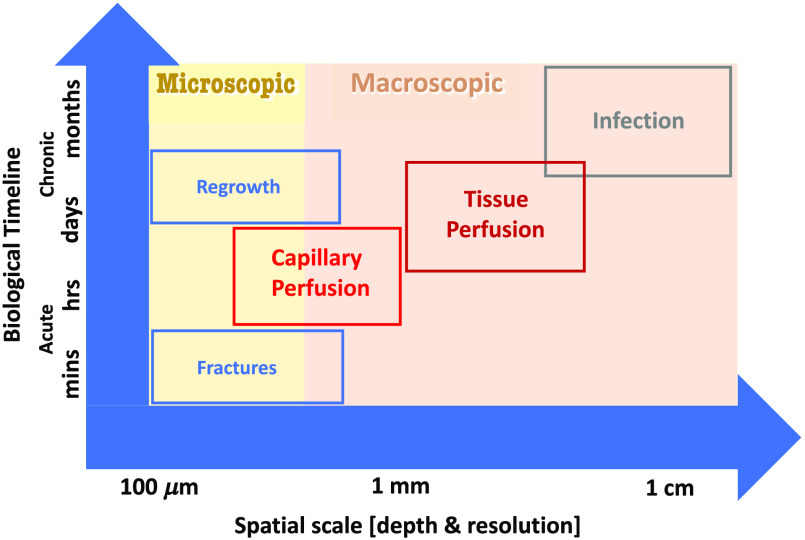
The matching process of the capabilities of each optical tool with the needs in each application.

Figure 1 shows an overview of the needs in trauma surgery, based upon the depth of penetrance of the imaging tool and the biological timeline of the disease. This figure provides a basic template from which to think about the tools and their value to trauma or disease diagnostics. While fine structured fractures might be seen with high-resolution tools, this is the realm of x-ray imaging or microscopy with optics such as OCT.^91^^,^^92^ Vascular function such as flow or perfusion to the tissue can be assessed microscopically or macroscopically, based upon the scope of the problem, but most clinical evaluations would require macroscopic sensing such as with NIRS, fluorescence, or photoacoustics. Biological functions such as regrowth or necrosis have two very different sampling scales, either microscopic methods such as UV and antibiotic fluorescence or macroscopic tools such as NIRS and contrast agent fluorescence.^27^^,^^44^^,^^45^^,^^49^ Again, for most clinical issues, the sampling of macroscopic signals is likely needed, and the combination with existing clinical tools such as ultrasound or x-ray would be beneficial.

## 5 Conclusions

Research is needed to optimize optical imaging techniques for tissue trauma surgical applications, and the developments described in Sec. 4 are each important to test. Based on the safety profile and previous implementation success to other surgical domains, the barriers to the translation of these techniques to orthopaedic surgery are relatively low. We believe that using optical imaging to provide surgeons with real-time objective data regarding bone and tissue perfusion will lead to more effective patient-specific management of common orthopaedic conditions with lower morbidity and will result in decreased variability associated with how these conditions are managed. Information provided should go beyond the basic soft and hard tissue structures available with x-ray imaging and incorporate functional flow and perfusion or tissue metabolism features that are likely to have a higher specific correlation to the outcome of the procedure. Optical tools have the best opportunity to impact surgery because of their inherent point-of-care use, their relatively low capital costs, and their compatibility with intraprocedure measurement.
